# Identification of Phthalates from Artificial Products in Chinese Kindergarten Classrooms and the Implications for Preschool Children’s Exposure Assessments

**DOI:** 10.3390/ijerph19138011

**Published:** 2022-06-30

**Authors:** Jiahui Wang, Zefei Xu, Jingyu Yao, Maochao Hu, Yuewen Sun, Cong Dong, Zhongming Bu

**Affiliations:** 1 School of Urban Construction, Hangzhou Polytechnic, Hangzhou 311402, China; jiahuiwang0921@163.com; 2 Department of Energy and Environmental System Engineering, Zhejiang University of Science and Technology, Hangzhou 310023, China; xzfei@163.com (Z.X.); yaojingyus@163.com (J.Y.); humc0407@outlook.com (M.H.); syw1003@126.com (Y.S.); lanyuanshishe@163.com (C.D.); 3 College of Energy and Environment, Shenyang Aerospace University, Shenyang 110136, China

**Keywords:** phthalates, di-2-ethylhexyl phthalate, emission source, Chinese kindergarten, children, exposure assessment

## Abstract

Phthalates are typical chemical pollutants in kindergarten classrooms since numerous artificial products (e.g., polyvinyl chloride (PVC) floorings, soft polymers and plastic toys) that might contain phthalates are widely distributed in kindergarten classrooms. Although Chinese preschool children spend a considerable amount of their waking hours (>8 h/day) in kindergartens, phthalate exposure in such indoor environment has not been given much attention. In this study, the mass fractions of six phthalates in twenty-six artificial products (fifteen flat decoration materials and eleven plastic toys) commonly found in Chinese kindergarten classrooms were measured. Di-2-ethylhexyl phthalate (DEHP) was the most predominant compound in all materials. The emission characteristics of the DEHP from these materials were further investigated. The measured emission characteristics were used for predicting multi-phase DEHP concentrations in kindergarten classrooms by applying a mass transfer model. The modeled concentrations were comparable with those measured in the real environment, indicating that these products might be the major sources of DEHP in Chinese kindergarten classrooms. Preschool children’s exposure to DEHP was found to be 0.42 μg/kg/day in kindergartens under baseline conditions, accounting for 18% of the total exposure to DEHP in Chinese indoor environments.

## 1. Introduction

Phthalates, known as plasticizers, are a group of emerging chemical pollutants in modern indoor environments [1,2]. Indoor phthalates originate from widely distributed source materials, such as soft polyvinyl chloride (PVC) and polymers, wallpapers, stickers, children’s toys and food packages [3,4]. Most phthalates are semi-volatile organic compounds (SVOCs) that exist in multiple phases indoors. Gaseous phthalates can be easily absorbed in airborne particles, settled dust and surfaces due to their low vapor pressure [2]. Humans are exposed to phthalates via inhalation, oral ingestion and dermal pathways [5,6,7,8,9]. Phthalates are rapidly metabolized after entering the human body and most phthalate metabolites are excreted in the urine [10,11,12,13]. This is of great concern owing to the associated toxicologic risks, such as endocrine disruption [14,15,16], reproductive system dysfunction [17,18,19], childhood asthma [20,21,22], neurodevelopmental disorders [23,24] and cancer [25].

The European Union and the US has been issuing regulations to restrict the usage of plasticizers in artificial products since the end of the last century [26,27,28]. However, the usage of plasticizers in China keeps growing due to rapid urbanization and modernization in the past few decades. In 2013, the production and consumption of plasticizers reached 4.3 and 2.45 million tons, respectively, accounting for 52% and 45% of global production and consumption [29]. Numerous phthalate-containing materials have been identified in the Chinese market, such as PVC floorings, wallpapers, window stickers and floor mats; the mass fractions of phthalates in those products could reach 10% or even greater [30,31]. Phthalates can be continuously released from those source materials, leading to the deterioration of indoor air quality. Previous studies have indicated that indoor phthalate concentrations in typical cities in China are at higher levels compared with those measured in other countries [32,33].

Children are more likely to be exposed to phthalates due to their exploratory behaviors [34], and their absorbed doses are usually higher than adults because they breathe or eat more per unit of body weight [35]. Bu et al. found that preschool children’s exposure to phthalates in Chinese indoor environments was roughly 80% higher than that for adults [32]. Previous studies have primarily focused on phthalate pollution in the Chinese home environment since it is the most important site for children’s indoor exposure [36,37,38]. Meanwhile, preschool children also spend many of their waking hours in daycare centers or kindergartens (e.g., 8–10 h per day, 5 days per week) [39]. However, research regarding phthalate pollution in daycare centers or kindergartens is limited in China. Recently, Wang et al. reported a median dust-phase level (the sum of 15 phthalates) in kindergarten buildings of 760 μg/g, which was higher than that measured in the home environment (i.e., 488 μg/g) in Beijing [40]. Another study found that the mean airborne concentrations of di(isobutyl) phthalate (DiBP), di-n-butyl phthalate (DnBP) and di-2-ethylhexyl phthalate (DEHP) in kindergarten classrooms in Beijing were 1.13, 0.93 and 0.22 μg/m^3^, respectively [41]. These reported values were comparable to or even higher than those measured in Chinese residences [33,36,42]. Given that a lot of synthetic decoration materials (e.g., PVC flooring, soft polymers, wall papers) and plastic toys can be found in kindergarten classrooms, the identification of phthalates from these artificial products is important to understand phthalate transfer and estimate children’s exposure in kindergartens.

Therefore, the objectives of the current study were to: (1) identify phthalates in twenty-six artificial products commonly found in Chinese kindergarten classrooms and investigate the emission characteristics of the phthalates in these materials; (2) estimate phthalate concentrations and try to explore major sources in kindergarten classrooms based on their emission characteristics and a mass transfer model; and (3) estimate preschool children’s exposure to phthalates in kindergarten classrooms based on modeled concentrations and investigate their potential contribution to total indoor exposures in China.

## 2. Materials and methods

### 2.1. Chemicals

Six common phthalates were selected as the target compounds, i.e., dimethyl phthalate (DMP), diethyl phthalate (DEP), DnBP, butyl benzyl phthalate (BBzP), DEHP, and di-n-octyl phthalate (DOP). A standard mixture of these chemicals (2000 mg/L of each phthalate in hexane) was purchased from the Organic Standard Solutions International Co., LLC, Columbia, SC, USA. Dichloromethane (DCM, TEDIA Co. Inc., Fairfield, OH, USA, HPLC grade) was used as the solvent for extraction.

### 2.2. Test Materials

**Figure 1:**
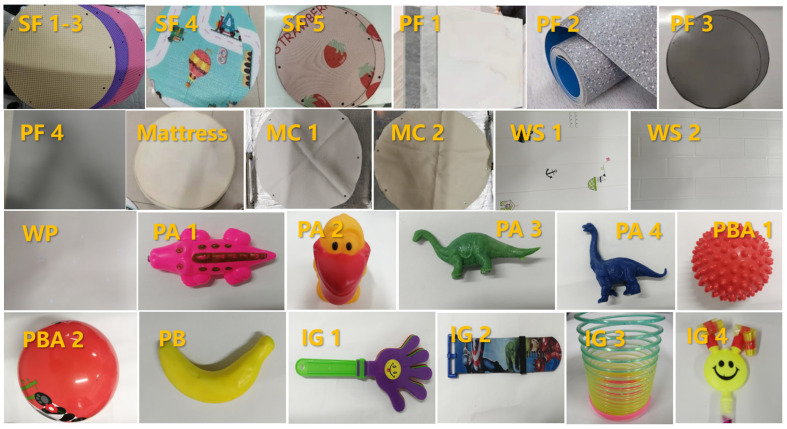
Photos of test materials in this study.

In this study, fifteen flat materials and eleven toys commonly found in kindergarten classrooms were selected as the test materials (shown in Figure 1). All the materials were purchased from a Chinese online shopping website. The flat materials included five soft floor mats (SF 1–5), four PVC flooring materials (PF 1–4), two mattress covers (MC 1 and 2), one mattress, two wall stickers (WS 1 and 2) and one wallpaper (WP). The toys were four plastic animals (PA 1–4), two plastic balls (PBA 1 and 2), one plastic banana (PB) and four other irregular shaped plastic toys (IG 1–4).

### 2.3. Identification of Phthalates from Test Materials

Three samples (approximately 0.3–0.8 g of each) were cut from random locations on each material. The samples were extracted separately with 60 mL of dichloromethane (DCM) at 70 °C for 6 h using a Soxhlet extractor. The extracts were concentrated into approximately 20 mL using a rotary evaporator, filtered through a 0.45 μm polytetrafluoroethylene (PTFE) microporous membrane, and then transferred into a Kuderna–Danish (K–D) tube. Thereafter, the clean extracts were further concentrated to 1 mL under a purified nitrogen stream. Finally, 200 μL of the concentrated extracts were transferred from the K–D tubes into 2 mL sample vials (Agilent Technologies, Part No. 5182-0553) equipped with 250 μL microvolume inserts. Final samples were stored at 4 °C in a laboratory refrigerator until analysis by a gas chromatograph–mass spectrometer (GC–MS) system (Agilent Technologies, Santa Clara, CA, USA, GC-7890N, MS-5975C). Detailed information on the chemical analysis by GC–MS is in Section S1 of the Supplementary Information (SI).

### 2.4. Measurement of Emission Characteristics of Phthalates from the Materials

Existing studies have concluded that phthalate emissions from solid materials can be characterized with a critical parameter: the gas-phase concentration immediately adjacent to the material surface (designated as *y*_0_, μg/m^3^) [43,44]. In this study, a solid-phase microextractor (SPME)-based sealed chamber method developed by Cao et al. [45] was applied to measure the *y*_0_ value of the flat materials at typical room temperature (25 °C), due to its short experimental time, the ease of the sampling procedure, and the simplicity of the experimental system. The *y*_0_ measurements were only conducted for DEHP because we found that DEHP was the most predominant phthalate in the material extraction experiments (see the results below). Details on the experimental procedure and method principle for determining *y*_0_ are provided in SI Section S2.

### 2.5. Estimation of Phthalate Concentrations in Kindergarten Classrooms

In our previous study, the transport of indoor SVOCs was described by a proposed mechanistic model [46]. Assuming that the transport of phthalates in the classroom has reached a steady state, the mass balance of gas- and particle-phase phthalates and suspended particles can be described as follows:

(1) $$\frac{\sum A_{e,i}h_{m}\left(y_{0,i}-C_{g}\right)}{V}-\alpha_{n}C_{g}-\alpha_{n}C_{sp}-v_{d}C_{sp}=0$$

(2) $$\frac{A_{sp}C_{p}h_{mp}}{\rho_{p}}\left(C_{g}-\frac{C_{sp}}{C_{p}K_{p}}\right)-\alpha_{n}C_{sp}-v_{d}C_{sp}=0$$

(3) $$\alpha_{n}PC_{p\_out}-\alpha_{n}C_{p}-v_{d}C_{p}=0$$

where *A_e_* (m^2^) is the surface area of the phthalate sources (the subscript “*i*” refers to the *ith* source material); *h_m_* (m/s) is the mass transfer coefficient above the source surface; *C_g_* (μg/m^3^) is the concentration of the gas-phase phthalate in the kindergarten classroom; *V* (m^3^) is the volume of the bedroom; *α_n_* (s^−1^) is the natural air exchange rate; *C_sp_* (μg/m^3^) is the concentration of the gas-phase phthalate in the classroom; *v_d_* (s^−1^) is the particle deposition rate constant; *A_sp_* (m^−1^) is the ratio between the surface area and the volume of a single particle; *C_p_* (μg/m^3^) is the mass concentration of suspended particles in the classroom; *h_mp_* (m/s) is the phthalate mass transfer coefficient between a single particle and air; *ρ_p_* (μg/m^3^) is the density of suspended particles; *K_p_* (m^3^/μg) is the partitioning coefficient between the particle- and gas-phase phthalate; *P* (-) is the particle penetration coefficient; and *C_p_out_* (μg/m^3^) is the mass concentration of suspended particles outdoors. As atmospheric phthalate concentrations were much lower than the indoor concentrations, the influence of atmospheric phthalates was not taken into account in the model. Phthalates also exist in indoor settled dust. The corresponding dust-phase concentration (*X_dust_*, μg/μg) can be estimated simply from the gas-phase by a linear equilibrium equation [47]:

(4) $$X_{dust}=C_{g}K_{dust}$$

where *K_dust_* (m^3^/μg) is the dust-air partitioning coefficient of a given phthalate. Combining Equations (1)–(4), the *C_g_*, *C_sp_*, *C_p_* and *X_dust_* in kindergarten classrooms at a steady state can be predicted.

The values of *y*_0_ in the flat source materials were based on our measurements as described in Section 2.4. For plastic toys, the corresponding *y*_0_ values were determined from the mass contents of given phthalates based on the method detailed by Cao et al. [45]. PM_10_ was considered to represent the outdoor suspended particles. One should be mindful that some particle-related parameters (*A_sp_*, *h_mp_*, *v_d_* and *P*) are related to particle size. For these parameters, we used integrated values (based on size-dependent values) to calculate airborne phthalate concentrations at a steady state. The outdoor PM_10_ was divided into 11 size bins according to Kawanaka et al.’s study [48]. The value for each size bin was first determined and then the integrated value was obtained by weighing the size-dependent concentrations by their mass fractions in the room air [30,31]. The size-dependent penetration coefficients of the outdoor particles were extracted from Liu et al.’s study [49]. The determination of the key parameters used in our calculations is further detailed in SI Section S3.

Indoor phthalate concentrations are related to some key environmental factors, such as the outdoor particle concentration (*C_p_out_*), air exchange rate (*a_n_*) and room temperature (*T*). Therefore, a sensitivity analysis was conducted to illustrate the variations in *C_p_out_*, *a_n_* and *T* on the modeled concentrations. Based on the annual concentration of outdoor PM_10_ in typical Chinese cities over the last five years, a baseline value of 80 μg/m^3^ was applied in the present study [46]. The lower and upper limits of *C_p_out_* were set to be 50 and 120 μg/m^3^, respectively, to represent an acceptable and heavily polluted level for the ambient air. The natural air exchange rate in the kindergarten classroom was set to be 1 h^−1^ under baseline conditions. The lower limit of *a_n_* was set to be 0.5 h^−1^ to reflect a poorly ventilated scenario and the upper limit was set to be 4 h^−1^ based on the average value recommended by the Chinese trade standard JGJ 39-2016, *Code for the design of nursey and kindergarten buildings* [50]. The range for the room temperature was set to be 16–28 °C according to the recommendations for conditioned spaces in the Chinese indoor air quality standard (GB/T 18883-2002) [51].

### 2.6. Children’s Exposure Assessments in Kindergarten Classrooms

In the present study, preschool children aged 1–5 years old were chosen as the target population. Inhalation, oral and dermal pathways were the typical exposure pathways during children’s indoor activities in kindergartens. For oral intake, only dust ingestion was considered since dietary ingestion was not included in the current study. For dermal pathways, only dermal absorption from the gas phase was considered. Children’s daily intakes via inhalation (*IE*, μg/kg/day), oral ingestion (*OE*, μg/kg/day) and dermal absorption (*DE*, μg/kg/day) were estimated based on the corresponding airborne concentrations at a steady state:

(5) $$IE=\frac{(C_{g}+C_{sp})\cdot IR_{inh}\cdot EF}{24\cdot BW}$$

(6) $$OE=\frac{X_{dust}\cdot IR_{dust}\cdot EF}{24\cdot BW}$$

(7) $$DE=\frac{C_{g}\cdot k_{p\_g}\cdot SA\cdot f_{s}\cdot EF}{24\cdot BW}$$

where *IR_inh_* (m^3^/day) is the inhalation rate; *EF* (h/day) is the children’s exposure frequency in kindergartens, i.e., 5.7 h/day for a week-long exposure (assuming 8 h/day on weekdays and zero on weekends); *BW* (kg) is the body weight; *IR_dust_* (g/day) is the dust ingestion rate; *k_p_g_* (m/day) is the transdermal permeability coefficient between air and dermal capillaries; *SA* (m^2^) is the total skin area; and *f_s_* (-) is the fraction of total skin exposed to room air. Detailed exposure factors for Chinese preschool children are listed in SI Table S1.

## 3. Results

### 3.1. Mass Fractions and Emissions of Phthalates in Test Materials

The mean values of the mass fractions in the test materials are listed in Table 1. Six target phthalates were detected in most of the artificial products except for DEP and BBzP. DEHP was the most abundant phthalate in both the flat materials and plastic toys, with a detection frequency of 100%. The results were in consistent with the fact that DEHP is currently the most frequently used plasticizer in China. Generally, the mass fractions of the target phthalates were not high (<1%) for all the materials. DEHP content was slightly higher in the flat materials than that in the plastic toys. For the flat materials, mass fractions in SF 5, MC 2 and PF 3 and 4 were at higher levels (0.37–0.74%). For plastic toys, phthalate contents were lower, except for DnBP in PBA 2 and DEHP in IG 2.

The *y*_0_ values of the five flat materials with the highest mass factions of DEHP (SF 5, PF 3 and 4, MC 3 and WP) were measured. The *y*_0_ values for the other flat materials could not be quantitively determined based on the SPME method due to their low DEHP content. As shown in Table 2, the mean *y*_0_ values of DEHP for the target flat materials were in the range of 0.14–0.30 μg/m^3^ at room temperature.

### 3.2. Modeled DEHP Concentratiosn in Kindergarten Classrooms

As shown in Table 3, the modeled gas- and particle-phase DEHP concentrations at a steady state under baseline conditions were 0.014 μg/m^3^ and 0.098 μg/m^3^, respectively. The corresponding dust-phase concentration was 840 μg/g. The variations in the air exchange rate and room temperature had a stronger impact on airborne DEHP concentrations than outdoor particle concentrations. The airborne DEHP concentration changed by 40–68% from its baseline value when the air exchange rate varied from 0.5 h^−1^ to 4 h^−1^ and the airborne concentration changed by 50–63% when the room temperature varied from 16 °C to 28 °C. Changes in the dust-phase DEHP levels were more sensitive to the outdoor particle concentrations and room temperature than the air exchange rate. The dust-phase DEHP concentration changed by 30–46% and 57–74%, respectively, when *C_p_out_* and *T* varied within their ranges.

### 3.3. Children’s Exposure to DEHP in Kindergarten Classrooms

**Figure 2:**
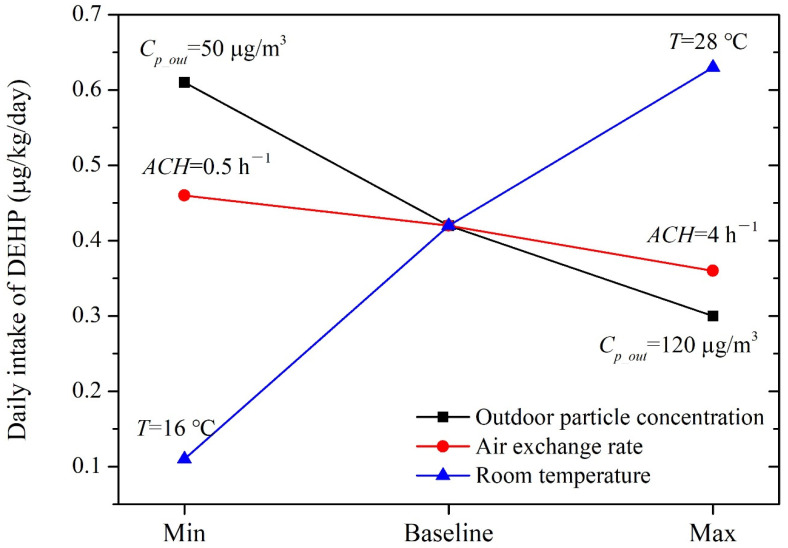
Sensitivity of the modeled DEHP exposure to key input parameters.

Under baseline conditions, children’s exposure to DEHP via three typical pathways was 0.42 μg/kg/day during daily activities in kindergarten classrooms. Exposure via dust ingestion contributed over 95% of the total intake. As shown in Figure 2, the estimated exposures were more sensitive to outdoor particle concentrations and room temperature than the air exchange rate. Children’s total daily intakes of DEHP varied within the ranges of 0.30–0.61 μg/kg/day and 0.11–0.63 μg/kg/day, respectively, when *C_p_out_* and *T* changed from the minimum to the maximum values. Children’s intakes changed from 0.46 μg/kg/day to 0.36 μg/kg/day when *a_n_* varied from 0.5 h^−1^ to 4 h^−1^ as the dust-phase concentration slightly changed with respect to variations in *a_n_*.

We compared these exposure estimates with children’s total daily intake in typical indoor environments to illustrate the potential contribution of exposure in kindergarten classrooms in China. Bu et al. reported a total daily intake of DEHP of 2.28 μg/kg/day (via inhalation, dust-ingestion and dermal absorption from the gas-phase) for children aged 1–5 years old [32]. Based on this value, it could be found that the exposure in kindergarten classrooms contributed roughly 18% under baseline conditions (with a range of 5–30%) to total indoor exposure to DEHP for preschool children in China.

## 4. Discussion

Previous studies have investigated the mass contents of phthalates in artificial products in Chinese indoor environments. For example, Shi et al. measured the mass contents of phthalates in 23 decoration materials in Chinese residences [31]. They found that DEHP was the predominant compound and that the highest mass fraction could reach 17%. Bu et al. reported phthalate levels in nine decoration materials in Chinese vehicle cabins and also found that the highest mass fractions were for DEHP (3–23%) [30]. These reported values were significantly higher than those observed for decoration materials or plastic toys in kindergarten classrooms in the present study. Currently, there are only two national standards (*Limit of Harmful Substances of Coatings for Toys* (GB 24613-2009) [52] and *Toys Safety* (GB 6675-2014) [53]) that provide guidelines for phthalate usage in children’s daily used products in China: i.e., the summed mass fraction of DnBP, BBzP and DEHP in a given toy or coating for toys should not exceed 0.1%. Based on this requirement, the phthalate contents (summed mass fraction of DnBP, BBzP and DEHP) in seven flat materials (SF 5, MC 3, WP and PF 1–4) and two toys (PBA 1 and IG 2; with a range of 0.14–0.95%) were higher than the threshold. Taken together, although the mass contents of the phthalates were relatively lower in the studied materials, more than one third of these artificial products used in kindergarten classrooms (at least in our measurements) did not meet the requirements specified in our national standards.

**Figure 3:**
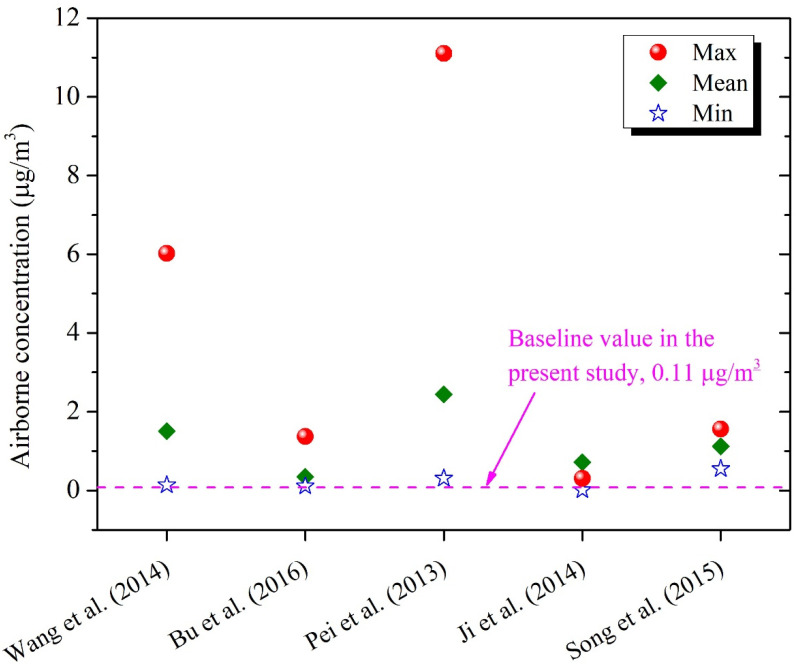
Comparison between our modeled DEHP concentrations and those measured in Chinese residences/offices. Only gas-phase concentrations were reported by Bu et al. [36], while airborne concentrations were reported for other studies [33,42,54,55].

We compared our predicted DEHP concentrations with those measured in kindergartens in China. Wang et al. reported airborne-phase and dust-phase DEHP concentrations for six kindergartens in three districts of Beijing, with ranges of 0.09–0.48 μg/m^3^ and 16.7–2240 μg/g, respectively (means: 0.22 μg/m^3^ and 330 μg/g) [41]. It was found that our estimates were generally comparable with those measured, indicating that these artificial products might be the major sources of DEHP in kindergarten classrooms in China. Wang et al. also reported considerable airborne concentrations of DMP and DnBP, i.e., 0.07–2.09 and 0.02–1.59 μg/m^3^, respectively [41]. However, the contents of these two phthalates were quite low in target materials based on our extraction experiments, indicating that there might be other potential sources of DMP and DnBP in Chinese kindergarten classrooms. Further research is required to identify the major sources of these lower molecular weight phthalates since they may also be used as solvents or carriers in consumer products, such as personal care products, varnishes or coatings [3,4]. Given that the parameters used in our model can hardly be consistent with those in the real environment, we acknowledged that the obtained data might not be enough to make a convincing comparison. We further compared our estimates with those measured for Chinese residences or office buildings (shown in Figure 3). The results indicated that airborne DEHP concentrations in kindergartens were lower than those in residential or office buildings [33,36,42,54,55], suggesting that DEHP pollution might be a more serious problem in residences or offices in China.

In the present study, a simplified model (with steady-state assumptions) was applied to describe the fate of phthalates in the classrooms. Moreover, when calculating inhaled doses, the desorption of particle-phase phthalates in the respiratory tract [56] was not considered. These simplified particle–gas interactions could lead to considerable uncertainties for inhalation exposure estimates [49,57,58]. For phthalates accumulated in settled dust, a linear partitioning behavior between the dust- and the gas-phase was considered. Recent studies have illustrated that a considerable percentage of phthalates could transfer from the source to dust if the dust is directly settled on the source surfaces [59,60,61], e.g., the PVC flooring and floor mats in our measurements. Given that oral ingestion contributed a large portion of children’s DEHP exposure indoors, the ignorance of direct transfer from source to dust could lead to considerable underestimation of exposure estimates. On the other hand, phthalate transfer from both source and sink surfaces to hands via children’s surface touch behaviors was not considered. This would consequently result in underestimation of daily intakes via both hand-to-mouth contact [62,63,64] and dermal absorption [65,66,67]. In addition, the impact of clothing on children’s dermal absorption [68,69] was not taken into account. Previous studies have indicated that dermal uptakes of phthalates could significantly increase when occupants wear phthalate-containing clothes [70,71,72]. Taken together, the modeled exposures in the present study might be a conservative estimation, indicating that Chinese children might be facing serious exposure to DEHP in kindergarten classrooms. In addition to refined phthalate transfer mechanisms and detailed exposure pathways (including surface touches), an internal exposure assessment approach (e.g., detection of metabolites in urine samples) could be helpful to improve the estimation of children’s exposure to phthalates in Chinese kindergartens.

## 5. Conclusions

Phthalates from twenty-six artificial products in Chinese kindergarten classrooms were identified. DEHP was the most predominant compound in all the materials. The gas-phase DEHP concentration immediately adjacent to the flat material surface was measured using an SPME-based method. Multi-phase DEHP concentrations in kindergarten classrooms were predicted based on the emission characteristics and a mechanistic model. The predicted DEHP concentrations were comparable with those measured in Chinese kindergartens, suggesting that these materials might be the major DEHP sources in kindergarten classrooms. The exposure estimates showed that preschool children’s exposure in kindergarten classrooms contributed 5–30% to the total DEHP exposure in typical indoor environments in China. The results indicate that the kindergarten classroom could be an important environment for preschool children’s indoor exposure to DEHP in China.

## Figures and Tables

**Table 1:** Mass fractions of target phthalates in test materials ^a^.

	DMP	DEP	DnBP	BBzP	DEHP	DOP
*Flat materials*						
Floor mat 1	/	nd	/	/	/	/
Floor mat 2	/	nd	/	/	/	/
Floor mat 3	/	nd	/	/	/	/
Floor mat 4	/	nd	/	/	/	/
Floor mat 5	/	nd	/	/	0.44%	/
PVC flooring 1	/	nd	/	nd	0.14%	0.08%
PVC flooring 2	/	nd	0.03%	/	0.22%	0.02%
PVC flooring 3	/	nd	0.08%	/	0.37%	/
PVC flooring 4	/	/	/	/	0.60%	/
Mattress	/	nd	/	/	/	/
Mattress cover 1	/	nd	/	/	/	/
Mattress cover 2	/	nd	/	/	0.74%	/
Wall sticker 1	/	nd	/	nd	0.03%	/
Wall sticker 2	/	nd	/	/	0.02%	/
Wall paper	/	nd	/	/	0.26%	0.02%
*Plastic toys*						
Animal 1	/	nd	/	nd	0.02%	/
Animal 2	/	nd	nd	nd	0.01%	/
Animal 3	/	nd	/	nd	/	nd
Animal 4	/	nd	/	nd	/	nd
Ball 1	nd	nd	0.95%	nd	/	/
Ball 2	/	nd	nd	nd	/	0.01%
Banana	nd	nd	/	nd	/	/
IG 1	/	nd	/	nd	0.09%	/
IG 2	/	nd	0.04%	nd	0.22%	nd
IG 3	nd	nd	/	nd	/	nd
IG 4	/	nd	/	nd	/	/

^a^ “nd”: not detected; “/”: <0.01%.

**Table 2:** Measured *y*_0_ values of DEHP emitted from target flat materials.

	Mass Fraction	*y*_0_ (μg/m^3^)
Floor mat 5	0.44%	0.21 ± 0.03
PVC flooring 3	0.37%	0.24 ± 0.01
PVC flooring 4	0.60%	0.29 ± 0.01
Mattress 3	0.74%	0.30 ± 0.02
Wall paper	0.26%	0.14 ± 0.01

**Table 3:** Modeled DEHP concentrations in kindergarten classrooms and sensitivity analysis.

	Gas Phase (μg/m^3^)	Particle Phase (μg/m^3^)	Dust Phase (μg/g)
Baseline	0.014	0.098	840
Outdoor particles			
* C_p_out_* = 50 μg/m^3^	0.020	0.089	1227
* C_p_out_* = 120 μg/m^3^	0.010	0.103	592
Air exchange rate			
* ACH* = 0.5 h^−1^	0.015	0.161	900
* ACH* = 4 h^−1^	0.012	0.027	735
Room temperature			
* T* = 16 °C	0.004	0.027	216
* T =* 28 °C	0.021	0.147	1322

## Data Availability

The data that support the findings of this study are available in the Supplementary Material of this article.

## Supplementary Materials

The following supporting information can be downloaded at: https://www.mdpi.com/article/10.3390/ijerph19138011/s1. Section S1, Detailed information on the chemical analysis by the GC–MS system; Section S2, Detailed information for measuring the *y*_0_ values of DEHP; Section S3, Parameters for estimating DEHP concentrations in kindergarten classrooms; Table S1, Exposure factors for preschool children (1–5 years old) in urban China; Figure S1, Size distribution of outdoor particles. References [73,74,75,76,77,78,79] are cited in the Supplementary Materials.
