# Supplementary material for: Identification of Phthalates from Artificial Products in Chinese Kindergarten Classrooms and the Implications for Preschool Children’s Exposure Assessments
Source: Int J Environ Res Public Health. 2022 Jun 30;19(13):8011. doi: 10.3390/ijerph19138011 (PMC9265414; doi:10.3390/ijerph19138011)
Supplement: Supplementary file 1 [file ijerph-19-08011-s001.zip › ijerph-1724118-supplementary.pdf]

Supporting Information **for**

Identification of Phthalates from Artificial Products in Chinese Kindergarten Classrooms and the Implications for Preschool Children's Exposure Assessments

Jiahui Wang [1], Zefei Xu [2], Jingyu Yao [2], Maochao Hu [2], Yuewen Sun [2,3], Cong Dong [2] and Zhongming Bu [2,*]

1 School of Urban Construction, Hangzhou Polytechnic, Hangzhou 311402, China; jiahuiwang0921@163.com
2 Department of Energy and Environmental System Engineering, Zhejiang University of Science and Technology, Hangzhou 310023, China; xzfei@163.com (Z.X.); yaojingyus@163.com (J.Y.); humc0407@outlook.com (M.H.); syw1003@126.com (Y.S.); lanyuanshishe@163.com (C.D.)
3 College of Energy and Environment, Shenyang Aerospace University, Shenyang 110136, China
* Correspondence: zmbu@zust.edu.cn

Section S1. Detailed information of chemical analysis by GC-MS system

All samples, including the solvent extracts and solid-phase microextractor (SPME) samples, were analyzed by a gas chromatography-mass spectrometry (GC-MS). A chromatographic column with structure of 30m×0.25mm×0.25μm (HP-5MS, Helium gas at 1.0 mL/min) was used for chromatography separation. Mass spectrometry was operated in both Scan and SIM (Selected Ion Monitoring) modes. The targeted compounds were quantified by the selected molecular ions: m/z = 163 for dimethyl phthalate (DMP) and m/z = 149 for the other phthalates. The temperatures of the injection port and ion source was 280 °C and 250 °C, respectively. The GC oven temperature was maintained at 80 °C for 2 min, increased to 220 °C at 10 °C/min and maintained for 3 min, and further increased to 300 °C at 20 °C/min and maintained for 3 min. For solvent extracts, 1 μL extracts were injected into the GC injection port. SPME was directly inserted into the GC injection port and maintained for 5 min. Phthalates sorbed on SPME were thermally desorbed (at 280 °C) and transferred to the chromatographic column.

A nine-point calibration curve was obtained by 1 μL injection of standard mixture with concentrations of 0.05, 0.1, 0.2, 0.5, 1.0, 2.0, 5.0, 10 and 20 μg/mL (of each phthalate). A linear function between the

peak area and the injected amount of each phthalate was assumed valid when R^2 of the linear function was greater than 0.99. For solvent extracts, the detection range was 0.05-20 μg/mL. Phthalate concentrations in the extraction lab blanks were all lower than the detection limit of quantification (LOQ). For the SPME samples, the detection range was 0.05-20 ng (based on the injection volume of 1 μL).

Section S2. Detailed information for measuring y_0 values of DEHP

1) Experimental system

Detailed information about the experimental system can be found in Cao et al.[1] Two circular samples (diameter: 44.0 cm) were cut from random locations of each material, which were placed on the top and bottom of a circular ring made of stainless steel (thickness: 2.0 cm; inner diameter: 40.0 cm; outer diameter: 44.0 cm). Eight through-holes were uniformly distributed on the side of the circular ring so that SPME could be inserted into the chamber. Two flat plates made of stainless steel (thickness: 5.0 mm; diameter: 44.0 cm) were then placed on the other side of each sample. Thereafter, the chamber was sealed by pressing the two flat plates together using several bolts. The temperature of the whole chamber was conditioned by a water bath system with a precision of 0.5°C. When sampling the phthalates in the chamber air, SPME (Supelco Analytical, Cat. No. 57302, Sigma-Aldrich Co. LLC) was inserted into the chamber to let SPME be exposed to the chamber air. Phthalates in the chamber air was then sorbed by SPME. After a certain sampling time, SPME was taken out from the chamber and the sampling procedure was completed. The amount of the targeted phthalates sorbed on SPME fiber was then quantified using the GC-MS system (see details in SI Section S1).

2) Conditioning of SPME before experiments

Before each measurement, SPME was inserted into the GC injection port and thermally desorbed at 280 °C for 5 min to ensure that the mass of phthalates of the remaining in the coating were below the LOQ. Our measurements indicated that no phthalates can be detected in SPME after one-time conditioning.

3) Method principle for determining y_0

Based on the measuring procedure for y_0, the sampling time would fall in the period that the amount of phthalate is linearly related with the sampling time [1]. Based on existing studies, the critical time for the linearly increasing period, designated as t_{linear}, is defined as the period during which the sorbed amount is less than 50% of the equilibrium amount [2]. When the sampling time is shorter than t_{linear}, y_0 could be determined as follows [1]:

$$y_0 = \frac{M}{D_a \cdot S \cdot t} \tag{S1}$$

where M (μg) is the amount of phthalate sorbed on SPME; D_a (m^2/s) is the diffusion coefficient of phthalate in the air (determined as 3.37×10^{-6} m^2/s for DEHP at 25°C [3]); t (s) is the sampling time; and S (m) is the shape factor, which is determined by the following equation:

$$S = 4\pi H\sqrt{1-\gamma^2} \Big/ \ln\left[\frac{1+\sqrt{1-\gamma^2}}{1-\sqrt{1-\gamma^2}}\right], \quad \text{with} \quad \gamma = \frac{d}{H} \tag{S2}$$

where H (m) is the length of the SPME fiber coating and d (m) is the external diameter of the SPME fiber coating. For the SPME used in the present study, H = 0.01 m and d = 1.24 × 10^{-4} m. Thus, S equals 0.0124 m.

4) Determination of the sampling time of SPME

As we have pointed out in Section S1, M should fall in the range of 0.05-20 ng. Thus, the shortest sampling time (t_{min}) should meet the criteria that $M = D_a S y_0 t_{\min} > 0.05$ ng, i.e., $t_{min} > 0.05$ ng/($D_a S y_0$). The longest sampling time (t_{max}) should meet the criteria that $M = D_a S y_0 t_{\max} < 20$ ng, i.e., $t_{max} < 20$ ng/($D_a S y_0$). It should be noted that the value of t_{max} may exceed the value of t_{linear} if the value of y_0 is small. Thus, the sampling time of SPME should fall in the range of t_{min} to min(t_{max}, t_{linear}). Note: if t_{min} is greater than t_{linear}, the SPME method will not be applicable for measuring y_0.

Based on previous studies using the same SPME in the present study, the value of t_{linear} for DEHP sampling was approximately 20 h [1,4]. In the present study, the sampling time for DEHP was set to be 8 h at 25 °C, to ensure that the sampling time fall in the range of t_{min} to min(t_{max}, t_{linear}).

Section S3. Parameters for estimating DEHP concentrations in kindergarten classroom

1) y_0 value for different sources

In a typical Chinese kindergarten classroom, 5 types of emission sources were considered, i.e., PVC flooring, wall paper, floor mat and plastic toy. For the first four flat materials, the corresponding y_0 values at 25 °C were set by averaging the measured values for each source. For plastic toys, the y_0 value of DEHP at 25 °C was estimated based on the mass fraction in source materials [1]:

$$\frac{y_0}{y_{sat,DEHP}} = 1 - e^{-8.6m} \tag{S3}$$

where $y_{sat,DEHP}$ (μg/m^3) is the saturated gas-phase DEHP concentration at 25 °C, i.e., 2.6 μg/m^3 [1]; m (%) is the mass fraction of DEHP in a given plastic toy, which is set to be 0.15% based on our extraction experiments. Therefore, the y_0 value of DEHP emitted from plastic toys was determined to be 0.03 μg/m^3.

2) Dimension of a classroom and surface area of source materials

According to Chinese trade standard JGJ 39-2016, *Code for design of nursey and kindergarten buildings* [5], the minimum area of an activity room should be 35 m^2, and the minimum height should be 2.8 m. The ratio between window area and floor area should be 0.2. Assuming that the room floor is fully covered with PVC flooring and the wall is covered with wall papers, the surface areas of PVC flooring and wall paper are set to be 35 m^2 and 65 m^2, respectively. We further assumed a total area of 10 m^2 for floor mats in a single classroom. The total surface area of plastic toys in a kindergarten classroom is difficult to determine due to insufficient statistic data. As a conservative estimate, a baseline value of 10 m^2 was assumed. Sensitivity analysis indicated that the variation of total surface

area of plastic toys had little influence on indoor DEHP concentrations since the emission strengths of plastic toys were quite low based on our extraction experiments.

3) Partitioning coefficients

The particle-air partitioning coefficient (K_p, m^3/μg) for DEHP was determined based on the equation given by Weschler and Nazaroff [6],

$$K_p = \frac{f_{om\_part} K_{oa}}{\rho_p} \tag{S4}$$

where f_{om_part} (-) is the mass fraction of organic matters in particles (set to be 0.4); K_{oa} (-) is the octanol-air partitioning coefficient (K_{oa} of DEHP at 25°C were retrieved from Weschler and Nazaroff [6]); and ρ_p (μg/m^3) is the density of particles (set to be 1×10^{12} μg/m^3).

Dust-air partitioning coefficient (K_{dust}) for DEHP was set to be $10^{-1.21}$ m^3/μg based on Wei et al.'s study [7].

4) Deposition rate constant of suspended particles

According to Cao et al.[8], an empirical relationship between the deposition rate constant (v_d, s^{-1}) and particle diameter (d_p, μm) was used in the present study:

$$\begin{aligned}\log v_d =\ & 0.00911\left(\log d_p\right)^6 + 0.0952\left(\log d_p\right)^5 + 0.0190\left(\log d_p\right)^4 - 0.536\left(\log d_p\right)^3 \\ & + 0.327\left(\log d_p\right)^2 + 1.53\log d_p - 4.20\end{aligned} \tag{S5}$$

5) Mass-transfer coefficients

The convective mass-transfer coefficient above the material surface (h_m) is related to the air speed over the surface (v, m/s), which can be estimated as follows [9]:

$$\frac{h_{m,2}}{h_{m,1}} = \left(\frac{v_2}{v_1}\right)^{0.5} \tag{S6}$$

Shi and Zhao [10] estimated an h_m of 1.63 m/h based on an air speed over indoor surface of 0.15 m/s, which was treated as a baseline condition here. Thereafter, the correlation between h_m and v can be expressed as:

$$h_m = 1.63\left(\frac{v}{0.15}\right)^{0.5} \tag{S7}$$

Liu et al. [11] provided an empirical correlation between v and indoor air exchange rate (ACH, h^{-1}):

$$v = 0.15ACH^{0.15} \tag{S8}$$

Combining eqs. (S7) and (S8), the following can be obtained:

$$h_m = 1.63ACH^{0.075} \tag{S9}$$

According to Liu et al. [12], the particle-air mass transfer coefficient (h_{mp}, m/s) is a function of particle diameter (d_p, μm),

$$\begin{aligned} h_{mp} &= 0.554\left(\log d_p\right)^6 - 0.0168\left(\log d_p\right)^5 - 4.54\left(\log d_p\right)^4 + 0.981\left(\log d_p\right)^3 \\ &\quad + 11.1\left(\log d_p\right)^2 - 14.0\log d_p + 6.60 \end{aligned} \tag{S10}$$

6) Integrated size-related parameters of indoor particles

The integrated parameters were determined by weighing the size-dependent ones by their mass fractions (f_p) in the room air. The values of f_p could be calculated based on the mass-balance of suspended particles within each size bin at steady state. Taking v_d as an example, the integrated v_d ($v_{d,int}$) of particles in the room air was calculated as follows:

$$v_{d,\text{int}} = \sum_{i=1}^{11} f_{p,i} v_{d,i} \tag{S11}$$

where i represents the ith size bin. For each size bin, the representative value (e.g., $v_{d,i}$) was set as the value corresponding to the upper limit of the diameter. The size distribution of ambient particles is shown in Figure S1.

Table S1. Exposure factors for preschool children (1~5 years old) in urban China[a]

Parameters	Values
Inhalation rate, *IR* (m^3/day)	7.2
Body weight, *BW* (kg)	14.9
Skin surface area, *SA* (m^2)	0.65
Fraction of skin exposed to air, f_s (-)	0.3
Transdermal permeability coefficient, k_{p_g} (m/day)	
DEHP	139.2

[a] Values were all derived from Bu et al. (2019) [13].

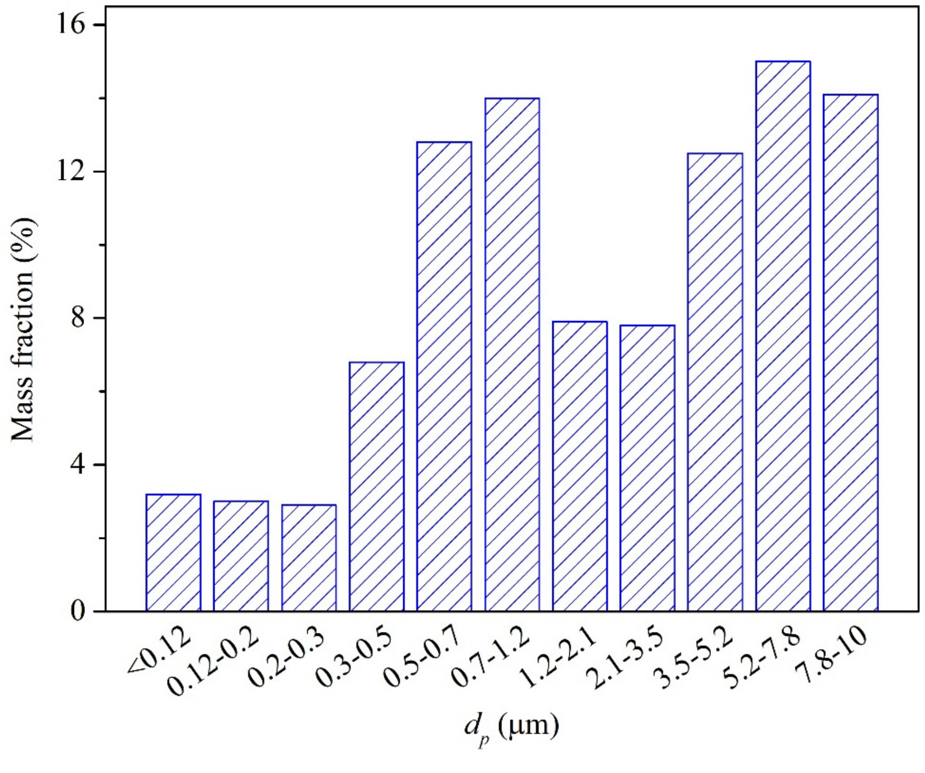

Figure S1. Size distribution of outdoor particles [14].

References

1. Cao, J.; Zhang, X.; Little, J.C.; Zhang, Y., A SPME-based method for rapidly and accurately measuring the characteristic parameter for DEHP emitted from PVC floorings. *Indoor Air.* **2017**, *27* (*2*), 417-426.
2. Ouyang, G.; Pawliszyn, J., A critical review in calibration methods for solid-phase microextraction. *Anal. Chim. Acta.* **2008**, *627*, 184-197.
3. Lugg, G.A., Diffusion coefficients of some organic and other vapors in air. *Anal. Chem.* **1968**, *40* (*7*), 1072-1077.
4. Shi, S.; Cao, J.; Zhang, Y.; Zhao, B., Emissions of Phthalates from Indoor Flat Materials in Chinese Residences. *Environ. Sci. Technol.* **2018**, *52* (*22*), 13166-13173.
5. JGJ39-2016, Code for design of nursey and kindergarten buildings. In Beijing: China Architecture & Building Press, 2016.
6. Weschler, C.J.; Nazaroff, W.W., SVOC partitioning between the gas phase and settled dust indoors. *Atmos. Environ.* **2010**, *44* (*30*), 3609-3620.
7. Wei, W.; Mandin, C.; Blanchard, O.; Mercier, F.; Pelletier, M.; Le Bot, B.; Glorennec, P.; Ramalho, O., Distributions of the particle/gas and dust/gas partition coefficients for seventy-two semi-volatile organic compounds in indoor environment. *Chemosphere.* **2016**, *153*, 212-9.
8. Cao, J.; Mo, J.; Sun, Z.; Zhang, Y., Indoor particle age, a new concept for improving the accuracy of estimating indoor airborne SVOC concentrations, and applications. *Build. Environ.* **2018**, *136*, 88-97.
9. Axley, J.W., Adsorption modelling for building contaminant dispersal analysis. *Indoor Air.* **1991**, *2*, 147-171.
10. Shi, S.; Zhao, B., Estimating indoor semi-volatile organic compounds (SVOCs) associated with settled dust by an integrated kinetic model accounting for aerosol dynamics. *Atmos. Environ.* **2015**, *107*, 52-61.
11. Liu, C.; Zhang, Y.; Benning, J.L.; Little, J.C., The effect of ventilation on indoor exposure to semivolatile organic compounds. *Indoor Air.* **2015**, *25* (*3*), 285-96.
12. Liu, C.; Zhang, Y.; Weschler, C.J., The impact of mass transfer limitations on size distributions of particle associated SVOCs in outdoor and indoor environments. *Sci. Total Environ.* **2014**, *497-498*, 401-11.
13. Bu, Z.; Mmereki, D.; Wang, J.; Dong, C., Exposure to commonly-used phthalates and the associated health risks in indoor environment of urban China. *Sci. Total Environ.* **2019**, *658*, 843-853.
14. Kawanaka, Y.; Tsuchiya, Y.; Yun, S.; Sakamoto, K., Size distributions of polycyclic aromatic hydrocarbons in the atmosphere and estimation of the contribution of ultrafine particles to their lung deposition. *Environ. Sci. Technol.* **2009**, *43*, 6851-6856.
